# Tumor Cell Capture Using Platelet-Based and Platelet-Mimicking Modified Human Serum Albumin Submicron Particles

**DOI:** 10.3390/ijms232214277

**Published:** 2022-11-18

**Authors:** Xiaotong Zhao, Radostina Georgieva, Pichayut Rerkshanandana, Moritz Hackmann, Lara-Elena Heil Olaizola, Maxine Müller-de Ahna, Hans Bäumler

**Affiliations:** 1 Institute of Transfusion Medicine, Charité-Universitätsmedizin Berlin, 10117 Berlin, Germany; 2 Department of Medical Physics, Biophysics and Radiology, Medical Faculty, Trakia University, 6000 Stara Zagora, Bulgaria

**Keywords:** human serum albumin (HSA), platelets, circulating tumor cells, submicron particles, adhesion

## Abstract

The co-localization of platelets and tumor cells in hematogenous metastases has long been recognized. Interactions between platelets and circulating tumor cells (CTCs) contribute to tumor cell survival and migration via the vasculature into other tissues. Taking advantage of the interactions between platelets and tumor cells, two schemes, direct and indirect, were proposed to target the modified human serum albumin submicron particles (HSA-MPs) towards tumor cells. HSA-MPs were constructed by the Co-precipitation–Crosslinking–Dissolution (CCD) method. The anti-CD41 antibody or CD62P protein was linked to the HSA-MPs separately via 1-ethyl-3-(-3-dimethyl aminopropyl) carbodiimide hydrochloride (EDC) and N-hydroxysuccinimide (NHS) EDC/NHS chemistry. The size of modified HSA-MPs was measured at approximately 1 µm, and the zeta potential was around −24 mV. Anti-CD41-HSA-MPs adhered to platelets as shown by flowcytometry and confocal laser scanning microscopy. In vitro, we confirmed the adhesion of platelets to tumor lung carcinoma cells A549 under shearing conditions. Higher cellular uptake of anti-CD41-HSA-MPs in A549 cells was found in the presence of activated platelets, suggesting that activated platelets can mediate the uptake of these particles. RNA-seq data in the Cancer Cell Lineage Encyclopedia (CCLE) and The Cancer Genome Atlas (TCGA) database showed the expression of CD62P ligands in different types of cancers. Compared to the non-targeted system, CD62P-HSA-MPs were found to have higher cellular uptake in A549 cells. Our results suggest that the platelet-based and platelet-mimicking modified HSA-MPs could be promising options for tracking metastatic cancer.

## 1. Introduction

Hematogenous metastasis, a multi-step and complex process, is responsible for most cancer-related deaths [1]. Cancer cell spread begins when cancer cells acquire invasive potential, facilitating their escape from the primary tumor. The epithelial to mesenchymal transition (EMT) of tumor cells is considered to be the main driving force for cancer cell spread and CTC production [2,3,4]. After entering the vasculature, most CTCs die due to the hemodynamic forces, anoikis, and the immune system [5]. The surviving tumor cells exit the vasculature through endothelial cells [6]. 

Platelets can be activated by tumor cells and subsequently attach to the CTCs as a protective shield to help the CTCs survive in circulation [7,8,9]. The ATP released by tumor cell-activated platelets opens the endothelium barrier, allowing tumor cells to migrate transendothelially and, thereby, enhance cancer cell extravasation [10]. During the past decades, many studies have revealed receptor–ligand interactions between activated platelets and tumor cells, such as CD62P (P-selectin) and P-selectin ligands [11,12]. These findings lay the groundwork for the use of platelet-mediated delivery technology to treat cancer diseases. 

In view of the important role of platelets in tumor pathogenesis, there is a growing interest in utilizing platelets to develop efficient anti-tumor therapeutic approaches. For instance, anti-PD-L1 antibodies were linked to the surface of platelets to treat breast carcinomas and leukemia [13,14]; covering particles with platelet membranes is another strategy that has been discussed [15,16]. However, platelets are easy to be activated and distorted during the manufacture of platelet carriers in vitro and increase the probability of forming a thrombus after re-transfusion. Platelets, similar to red blood cells, face restrictions in proper storage and contamination. Long-term maintenance of the biological functions of modified platelets is difficult. Furthermore, increased exogenous platelet numbers can result in tumor-related thrombosis and promote tumor progression [17]. Therefore, drug-loaded autologous platelets can be considered the ideal medium to deliver drugs to tumor cells. Mimicking some functions of platelets by protein particles could also be an excellent strategy to avoid the disadvantages caused by the exogenous platelet. 

Human serum albumin (HSA), the most abundant plasma protein, has been used as a multifunctional drug delivery platform in the biomedical field. The extensive research and application of HSA are attributed to its good biocompatibility, biodegradability, non-toxicity, and non-immunogenicity [18]. Some anticancer medications, including paclitaxel, Methotrexate, and 5-fluorouracil, are used successfully when coupled with albumin [19,20,21]. Abraxane^®^ (nanoparticle albumin-bound paclitaxel) for breast cancer treatment is already in clinical application [22,23]. Although HSA possesses great biological properties as a drug carrier, further research is still required to improve its selective targeting capability. Our team has developed a successful technique called Co-precipitation–Crosslinking–Dissolution (CCD) for the fabrication of protein particles [24,25,26]. Human serum albumin submicron particles (HSA-MPs) produced by this technique were already used as a vehicle platform successfully loaded with riboflavin and Doxorubicin [27,28]. 

**Figure 1:**
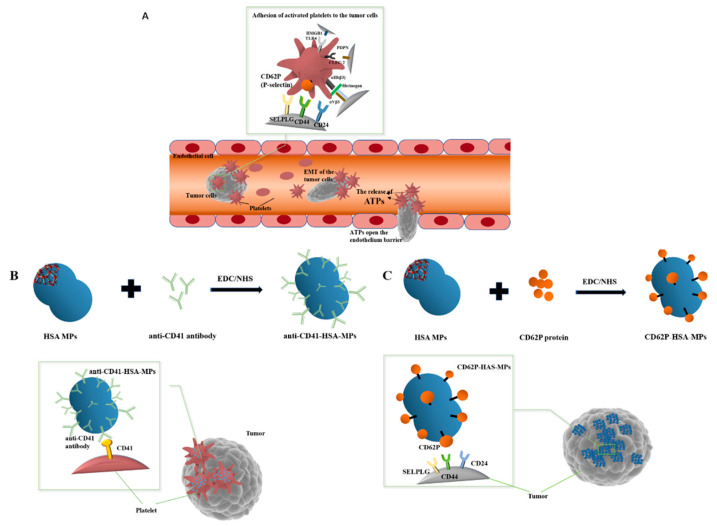
Schematic of targeting strategies. (**A**). Schematic of the circulating tumor cell captured by activated platelets in vivo, adhering and escaping through gaps of the endothelium. (**B**). Anti-CD41-HSA-MPs hitchhiked on platelets to target circulating tumor cells indirectly. (**C**). CD62P-HSA-MPs target a tumor cell directly mediated by the CD62P on the surface of the HSA-MPs.

In this study, we explored two drug delivery strategies that take advantage of the adhesion ability of platelets to CTCs (Figure 1A). (1) CD41, also known as GpIIb, is a cell-surface protein expressed at high levels on platelets. The anti-CD41 antibody-modified HSA-MPs are designed to trail tumor cells by hitchhiking on the platelet. (Figure 1B). (2) The mechanism of platelet aggregation around tumor cells includes the binding of biomolecules such as CD62P, which belongs to the selectin family. CD62P translocates to the platelet surface when platelets are activated, mediating the adhesion of platelets to the endothelial cells, leukocytes, and tumor cells [29,30]. We revealed the expression of common ligands for CD62P, including CD44, SELPLG, and CD24 [31], in pan-cancer analysis using TCGA and CCLE datasets. The CD62P enabled the HSA-MPs to specifically bind to the upregulated receptors on the surface of tumor cells. The platelet-mimicking particles with platelet-specific protein modifications, CD62P-HSA-MPs, were constructed to directly target tumor cells specifically (Figure 1C).

## 2. Results and Discussion

### 2.1. Characterization of Anti-CD41-HSA-MPs and CD62P-HSA-MPs

The size, polydispersity index (PDI), and zeta potential of anti-CD41-HSA-MPs and CD62P-HSA-MPs were analyzed by Zetasizer Nano ZS. Both particles showed a submicron size ranging from 0.9 to 1 µm, and the zeta potentials were around −24 mV (Table 1). When compared to reported HSA-MPs manufactured using the CCD method, the modified HSA-MPs’ sizes increased slightly [28,32,33].

### 2.2. Platelet Adhesion to the A549 Tumor Cells In Vitro

**Figure 2:**
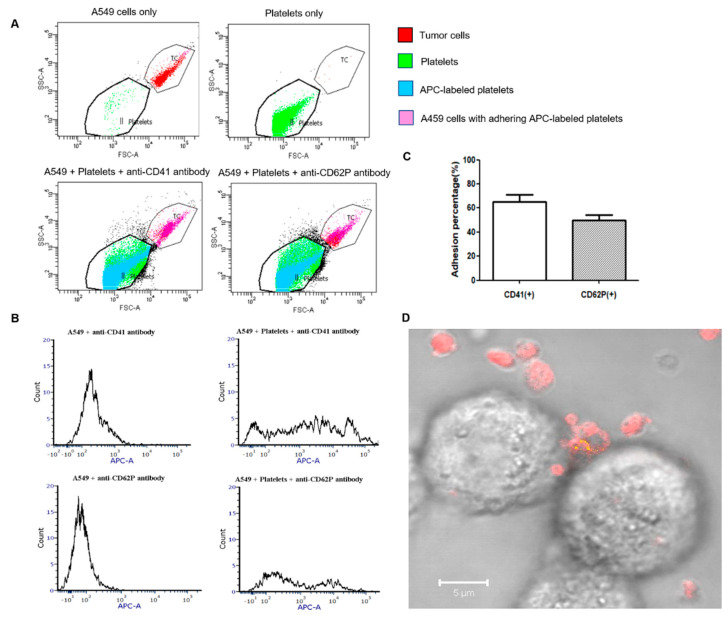
Adhesion of platelets to A549 cells under shearing conditions analyzed by flow cytometry and confocal laser scanning microscopy. (**A**). Dot plots of A549 cells, platelets, A549 cells + platelets + APC anti-CD41 antibody, and A549 + platelets + Alexa 647 anti-CD62P antibody (250 platelets/tumor cell). The platelets were labeled with APC anti-CD41 antibody or Alexa 647 anti-CD62P antibody separately. The A549 cell and platelet populations were identified by their forward scatter (FSC) and side scatter (SSC). (**B**). Histograms of the fluorescence intensity in the tumor cell population for A549 cells + APC anti-CD41 antibody (control), A549 cells + Platelets + APC anti-CD41 antibody, A549 cells + Alexa 647 anti-CD62P antibody (control), and A549 cells + Platelets + Alexa 647 anti-CD62P antibody. (**C**). Percentage of A549 cells with adhering platelets labeled with APC anti-CD41 antibody or Alexa 647 anti-CD62P antibody. (**D**). Representative image of platelets adhering to A549 tumor cell as observed under the confocal laser scanning microscope. The platelets were labeled with APC anti-CD41 antibody (red fluorescence) and Alexa 488 anti-CD62P antibody (green fluorescence). The yellow color represents overlapped red and green fluorescence.

The adhesion ability of activated platelets to the CTCs was an essential basis in this study. Platelets were isolated from healthy humans and activated under shearing conditions using a rheometer to verify the adhesion between activated platelets and A549 cells. As shown In Figure 2A–C, the fluorescence intensity quantified by a flow cytometer in the APC channel also showed similar results. The percentage of A549 cells with APC anti-CD41 antibody-labeled or Alexa 647 anti-CD62P antibody-labeled platelets were 65 ± 11% and 50 ± 8%, respectively. The fluorescent images (Figure 2D) showed the adhesion of the platelet to the A549 tumor cell under the fluorescence microscope. The platelets were labeled with anti-CD41 antibody (red fluorescence) and the activated ones were labeled with Alexa 488 anti-CD62P antibody (green fluorescence). Many more platelets were labeled with the APC anti-CD41 antibody than with the Alexa 488 anti-CD62P antibody, probably due to the lower number of activated platelets and the fact that CD62P on the platelets itself acts as a binding site to the A549 cells.

Platelets are one of the first circulating cells that CTCs encounter during metastasis. Most CTCs only survive in circulation for a short time before being trapped in the endothelial cells of blood vessels or eliminated by the immune system. Under shear stress, approximately 0.1% of CTCs are physically protected by the platelet-rich thrombi surrounding them [34,35]. Activated platelets cover the CTCs as “coats” and prevent cancer cells from being eliminated, promoting cancer progression. The expression of four platelet activation markers, including CD62P, GPIIb/IIIa, lysosomal glycoprotein, and phosphatidylserine, significantly increases following transitory exposure to higher shear strain rates [36]. In vitro, we confirmed the adhesion of platelets to the A549 cells under shearing conditions through flow cytometry and microscopy. This process can be explained by the activation of platelets under shear stress and the translocation of the tumor cell receptors, such as CD62P, to the activated platelet surface, which mediates the adhesion of platelets and tumor cells.

### 2.3. Anti-CD41-HSA-MP Adhesion to Platelets In Vitro

**Figure 3:**
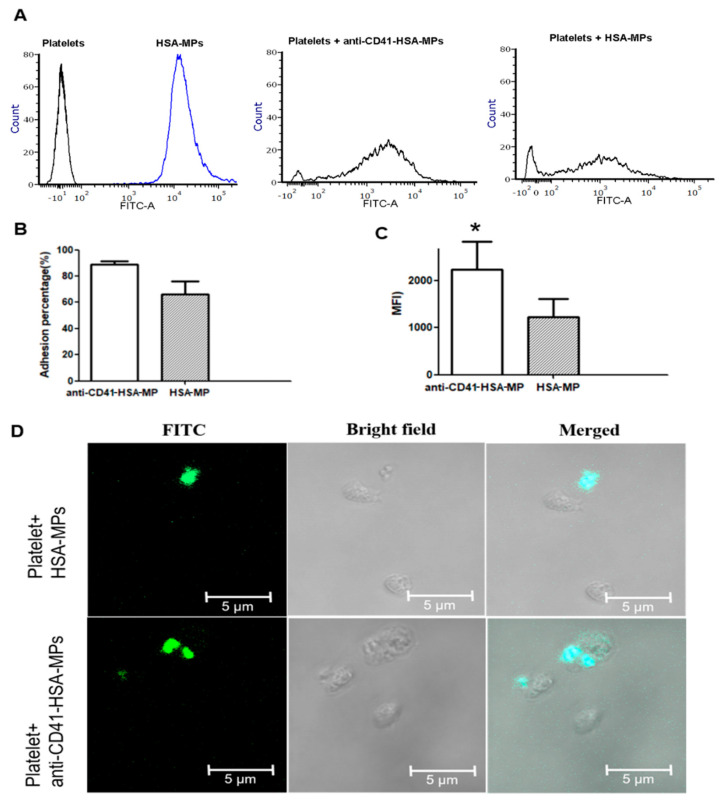
Adhesion of anti-CD41-HSA-MPs to platelets (**A**). Flow cytometry histograms of the fluorescence intensity in the platelet or FITC-HSA-MP populations (black line: platelets only; blue line: FITC-HSA-MPs only), and in the populations of platelets incubated with FITC-HSA-MPs or platelets incubated with anti-CD41-FITC-HSA-MPs, respectively. (**B**). Percentage of platelets with adhered FITC-HSA-MPs or anti-CD41-FITC-HSA-MPs. (**C**). Mean fluorescent intensity (MFI) values of the platelet population in the FITC channel. * *p* < 0.05. (**D**). Representative confocal laser scanning microscopy images of HSA-MPs and anti-CD41-HSA-MPs with platelets.

The results of the adhesion experiments performed by incubating platelets with the FITC-labeled HSA-MPs (20 particles/platelets) for 30 min are summarized in Figure 3. The flow cytometry results showed that the adhesion percentage of anti-CD41-FITC-HSA-MPs to the platelets was slightly higher than that of non-modified control FITC-HSA-MPs (Figure 3B). The higher mean fluorescence intensity of platelets in the FITC channel (Figure 3C) and typical fluorescence microscope images (Figure 3D) indicated that more anti-CD41-HSA-MPs adhere to the A549 cells than control HSA-MPs. 

It should be noted that the number of activated platelets is small. The majority of platelets in natural blood circulation are resting platelets. Platelets can be activated in large numbers under certain conditions, such as disease or injury. CD41, a cell-surface protein, is highly expressed on platelets and is used as a biomarker for platelets. It is a good choice to regard CD41 as a platelet-binding target for HSA-MPs. It is more efficient to use the anti-CD41-HSA-MPs hitchhiking on platelets, which could be activated under pathological conditions, than hitchhike on activated platelets only. 

### 2.4. Cellular Uptake of Anti-CD41-HSA-MPs in A549 Cells Pre-Incubated with Platelets

Gp60, SPARC, Gp18, and Gp30 are the major albumin-binding proteins that are overexpressed in multiple tumors and the primary mechanism responsible for the uptake of albumin in tumors, a process that includes transcytosis through endothelium and endocytosis in tumor cells [37,38,39]. Because of the expression of albumin-binding proteins on the cell surface, the A549 cell line is a widely used tool for studying the interaction of albumin particles and tumor cells [27,33,40]. It has already been shown that the HSA-MPs can bind to and be endocytosed by A549 cells [27].

**Figure 4:**
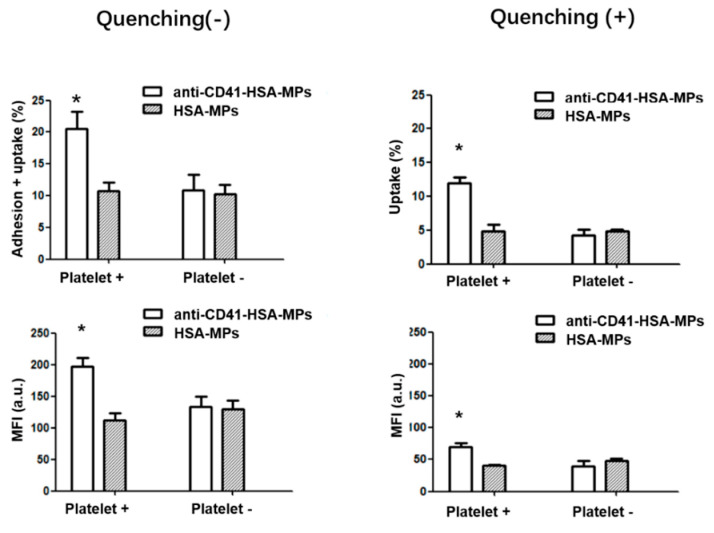
Adhesion vs. cellular uptake of anti-CD41-HSA-MPs and HSA-MPs in A549 cells analyzed by flow cytometry with or without pre-incubation with activated platelets. Adhering particles are excluded by quenching with Trypan blue. The upper graphs show the percentage of events in the A549 cell population with enhanced fluorescence in the FITC channel. The lower graphs show the corresponding Mean Fluorescence Intensities. * *p* < 0.05.

A549 cells were pre-incubated with arachidonic acid-activated platelets for 30 min and then FITC-labeled anti-CD41-HSA-MPs or HSA-MPs were added and the samples were incubated for 24 h. The fluorescence intensity analyzed by the flow cytometer (Figure 4) showed a significantly higher cellular uptake of anti-CD41-HSA-MPs than that of HSA-MPs in the presence of platelets. In the absence of the platelets, the cellular uptake of anti-CD41-HSA-MPs was reduced and had no difference with HSA-MPs. Measurement of fluorescence using flow cytometry allowed us to determine the A549 cells with internalized or adherent particles. The fully internalized particles were then retained by quenching the fluorescence of the remaining extracellular particles using Trypan blue. The cellular uptake percentage and MFI value decreased in all groups after quenching. However, the cellular uptake of anti-CD41-HSA-MPs in the presence of platelets was still significantly higher than that of HSA-MPs, which means that the platelet-adhering anti-CD41-HSA-MPs could be internalized more efficiently by A549 cells than the HSA-MPs in the presence of activated platelets. 

The results could be explained by platelets serving as a connection between anti-CD41-HSA-MPs and tumor cells. The activated platelets could adhere to tumor cells. Hitchhiking of HSA-MPs on platelets via anti-CD41 promotes the possibility of particle/tumor cell interaction due to the adherence of activated platelets to the cells. The internalization of the anti-CD41-HSA-MPs is then further supported through the albumin-binding receptor proteins on the surface of the tumor cells. This means that the anti-CD41-HSA-MPs could detect the tumor cells with the help of platelets in the circulation and could be used to deliver anticancer drugs, avoiding an ex vivo treatment of platelets.

### 2.5. Cellular Uptake of CD62P-HSA-MPs in A549 Cells

**Figure 5:**
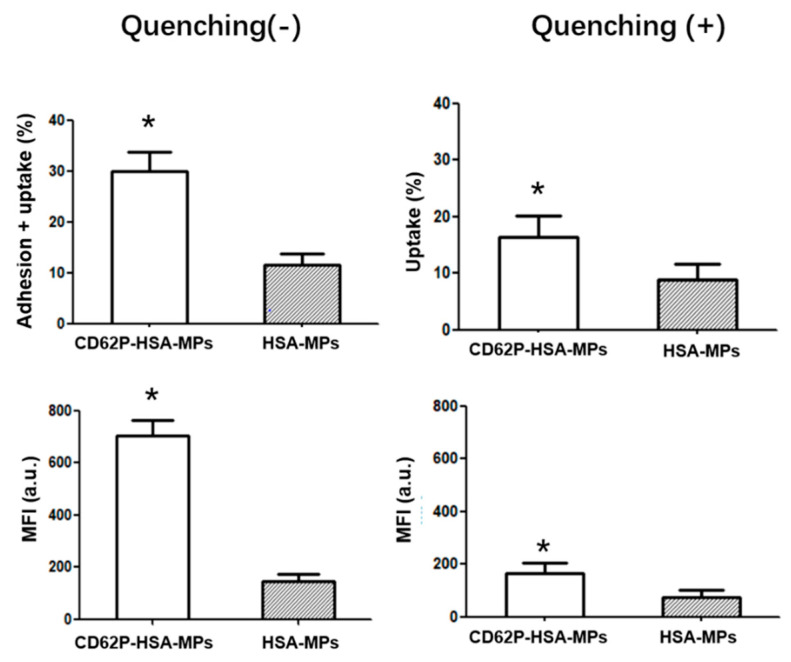
Adhesion vs. cellular uptake of anti-CD62P-HSA-MPs and HSA-MPs in A549 cells analyzed by flow cytometry. Adhering particles are excluded by quenching with Trypan blue. Upper graphs show the percentage of events in the A549 cell population with enhanced fluorescence in the FITC channel. Lower graphs show the corresponding Mean Fluorescence Intensities. * *p* < 0.05.

A549 cells were incubated with FITC-labeled CD62P-HSA-MPs or HSA-MPs for 24 h, and the resulting fluorescence per cell was measured by flow cytometry after quenching or not. The HSA-MPs served as a control. The cellular uptake percentage and MFI in the CD62P-HSA-MP group were both higher than those in the HSA-MP group with and without quenching (Figure 5). This means that CD62P-HSA-MPs could be internalized more efficiently than the HSA-MPs by the A549 cells. 

The CD62P-HSA-MPs are expected to be used to specifically affect the formation of platelet “coat” through competitive inhibition. These results help us further in the next step to develop a targeted and sustained drug delivery system. We realize that the interactions between targeted drug carriers and cancer cells may differ in a physiological experimental environment, such as in the presence of other natural blood cells. It may be necessary to perform in vivo tests in the future to explore the effects of the modified HSA-MPs in the circulation.

### 2.6. The Expression Level of the Ligands to CD62P in CCLE and TCGA Databases

CD62P has been suggested as an essential molecule for the adhesion of platelets to tumor cells. CD62P ligands, α_IIb_β_3_ integrins–fibrinogen-α_V_β_3_, CLEC-2–PDPN, and TLR4–HMGB1 are other receptor–ligand interactions that support the activation of platelets and aggregation on tumor cells [41,42,43]. Compared to the other receptor–ligand interactions, the related research on CD62P and its ligands is more extensive and in-depth. Gong et al. demonstrated that CD62P expression on platelets in the peripheral blood of cancer patients was significantly higher than that in healthy patients [44]. Moreover, different kinds of CD62P ligands, such as CD44, SELPLG, and CD24, have been shown overexpressed in different human carcinomas [31,45,46,47,48,49,50]. In practical drug delivery applications, tumor cells should be compared with noncancerous cells with low platelet-binding capacity when evaluating the platelet-mimicking carriers targeting tumor cells. In this instance, a high level of targeted binding with tumor cells is conducive to the use of a carrier system. 

**Figure 6:**
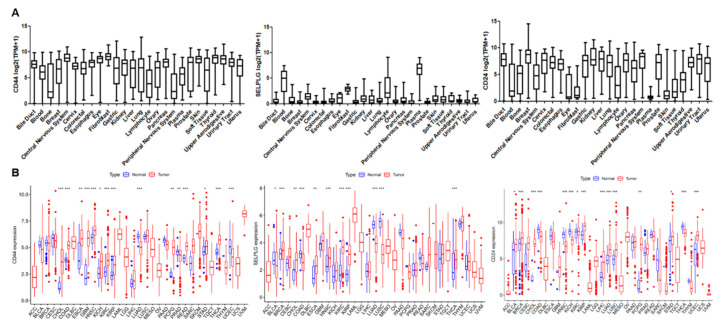
The mRNA expression of the ligands to the CD62P in the CCLE and TCGA database. (**A**). The mRNA expression levels of CD44, SELPLG, and CD24 in multiple cancer cell lines in the CCLE database. (**B**). The mRNA expression levels of CD44, SELPLG, and CD24 in multiple cancer types and corresponding normal tissues in TCGA database. * *p* < 0.05; ** *p* < 0.01; *** *p* < 0.001.

RNA-seq data in the CCLE database showed that CD44, SELPLG, and CD24 were expressed in almost all types of tumors (Figure 6A). Then, we retrieved the differential expression pattern of CD44, SELPLG, and CD24 in 33 types of cancers and adjacent normal tissues from the TCGA database. Our analysis showed that the expression of ligands was significantly higher in 17 cancers out of 33 types of cancers (Figure 6B). The above results provided a basis for the application of the CD62P-based albumin tumor-targeting carrier in tracking different types of CTCs.

## 3. Materials and Methods

### 3.1. Materials

APC anti-CD41 antibody, Alexa 647 anti-CD62P antibody, and Alexa 488 anti-CD62P antibody were purchased from BioLegend (San Diego, CA, USA). Anti-CD41 antibody (Biotin) was purchased from Antibodies.com (Cambridge, UK). CD62P protein was purchased from Sino Biological (Eschborn, Germany). Human serum albumin solution (200 g/L HSA, 145 mM NaCl) was from Grifols (Frankfurt, Germany). Dimethyl sulfoxide (DMSO) and sodium hydroxide (NaOH) were purchased from Carl Roth (Karlsruhe, Germany). Ethylene diamine tetra-acetic acid (EDTA) was purchased from Fluka (Buchs, Switzerland). Glutaraldehyde (GA), sodium carbonate (Na_2_CO_3_), magnesium chloride (MnCl_2_), phosphate-buffered saline (PBS), glycine, sodium borohydride (NaBH_4_), and fluorescein isothiocyanate (FITC) were purchased from Sigma-Aldrich (Munich, Germany). 1-Ethyl-3-(3-dimethyl aminopropyl) carbodiimide hydrochloride (EDC), 2-(N-morpholino)ethanesulfonic acid (MES), and (hydroxymethyl) aminomethane (Tris) were purchased from Thermo Scientific (Rockford, IL, USA). N-Hydroxysuccinimid (NHS) was purchased from Fluka (St. Louis, MO, USA). Arachidonic acid was purchased from Mölab (Langenfeld, German). 

### 3.2. Preparation of Human Serum Albumin Particles (HSA-MPs)

The HSA-MPs were fabricated by the CCD technique [24,27,28]. Briefly, equal volumes of 0.125 M MnCl_2_ with 10 mg/mL HSA and 0.125 M Na_2_CO_3_ were quickly added under stirring for 30 s at room temperature to produce HSA-MnCO_3_-MPs. According to the experimental needs, 0.025 mg/mL FITC in DMSO was added in the previous step. Then, 0.05% HSA solution was added to the suspension and incubated for an additional 5 min under stirring to prevent agglomeration of the particles. We then washed the HSA-MnCO_3_-MPs with 0.9% NaCl three times (3000 g, 5 min). The suspension was then cross-linked with 0.1% glutaraldehyde (GA) for 1 h at room temperature. Next, it was incubated with 0.08 M glycine and 0.625 mg/mL NaBH_4_ for 30 min to quench the remaining GA. Finally, 0.25 M EDTA was used to dissolve the MnCO_3_ templates (30 min). The obtained particles were washed three times (1000 g, 10 min) and stored in 0.9% NaCl for further use. 

### 3.3. Preparation of Anti-CD41-HSA-MPs and CD62P-HSA-MPs

**Figure 7:**
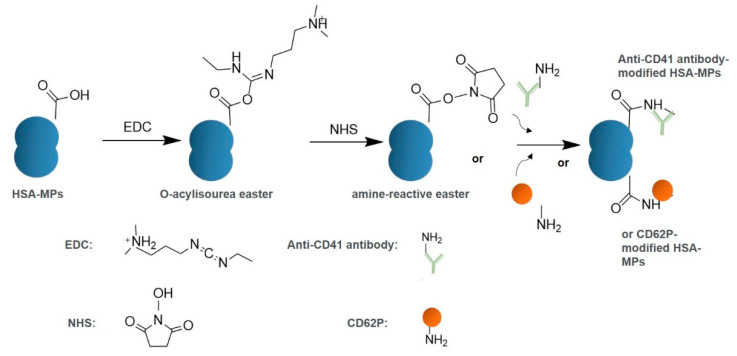
Scheme of modification on the surface of HSA-MPs by EDC/NHS method.

Anti-CD41 antibody was coupled to HSA-MPs through 1-ethyl-3-(-3-dimethyl aminopropyl) carbodiimide hydrochloride (EDC) and N-hydroxysuccinimide (NHS) EDC/NHS chemistry. First, HSA-MPs were repeatedly washed with activation buffer (50 mM MES buffer, PH 6). Then, 4.8 mM EDC and 48 mM NHS were simultaneously added to a 0.1% HSA-MP solution. This mixture was incubated at room temperature for 30 min. Afterwards, the anti-CD41 antibody was added to reach a 10 µg/mL concentration. We then mixed the solution well and then allowed the reaction to proceed for 2.5 h. Quenching was performed by adding glycine to a final 4 mg/mL concentration. Finally, the solution was washed with blocking buffer (50 mM Tris pH 8 and 0.5% HSA), and the final anti-CD41-HSA-MPs were stored in the blocking buffer. The same method was used during the preparation of CD62P-HSA-MPs (Figure 7).

### 3.4. Particle Characterization

The size, polydispersity index (PDI), and zeta potential of the anti-CD41-HSA-MPs and CD62P-HSA-MPs in 10 mM NaCl were measured by Dynamic Light Scattering (Zetasizer Nano ZS, Malvern Instruments Ltd., Malvern, UK). The results were expressed as mean ± standard deviation.

### 3.5. Cell Culture

The human pulmonary adenocarcinoma cell line A549 was a gift from Prof. Sergio Moya (CIC biomaGUNE, San Sebastian, Spain). The A549 cell line was cultured in RPMI 1640 medium (Corning, New York, NY, USA) supplemented with 10% FBS (Biochrom, Berlin, Germany) and 1% penicillin–streptomycin. The cells were incubated at 37 °C and 5% CO_2_ in the incubator (Thermo Scientific, Waltham, MA, USA).

### 3.6. Preparation of Platelets

Blood samples from healthy human volunteers (EA1/110/21—Ethics committee Charité) were collected into 0.105 M Na_3_ citrate tubes (366575, BD Vacutainer). The platelet-rich plasma (PRP) was isolated by centrifugation from whole blood (150× *g*, 15 min).

### 3.7. Adhesion of Platelets to Tumor Cells under Shearing Condition

Under normal hematological circumstances, the platelets activated in response to shear stress [51]. The high shear rates were able to induce platelet activation [36]. After trypsinization, A549 cells were incubated in PRP (tumor/platelet = 1/250) for 30 min under 3000 s^−1^ shear rate in a high-performance rheometer (Physica MCR 301, Anton Paar, Graz, Austria) at 37 °C. The platelets were marked with APC anti-CD41 antibody, Alexa 647 anti-CD62P antibody, or Alexa 488 anti-CD62P antibody. The percentage of cells with the adhesion of particles and the mean fluorescence intensity (MFI) of A549 cells were quantified by flow cytometry in the APC channel (BD FACS Canto II, Franklin Lakes, NJ, USA). The samples were also observed under a fluorescence microscope (CLSM ZeissLSM 510 meta, Zeiss MicroImaging GmbH, Jena, Germany).

### 3.8. Adhesion of Anti-CD41-HSA-MPs to Platelets

The PRP and anti-CD41-FITC-HSA-MP suspensions (20 particles/platelet) were mixed in the EP tube and incubated on the rotator at 37 °C. After 30 min of coincubation, the samples were fixed with 4% paraformaldehyde in PBS. After that, the adhesion of anti-CD41-FITC-HSA-MPs to platelets was analyzed using flow cytometry in the FITC channel and observed under the fluorescence microscope

### 3.9. Cellular Uptake Assay

In 24-well plates, A549 cells were seeded and incubated overnight. The medium was then replaced with fresh serum-free medium containing FITC-labeled HAS-MPs, anti-CD41-HAS-MPs, or CD62P-HSA-MPs (MPs/A549 = 5000/1) and co-incubated for 24 h. The activated platelets were prepared by incubating with arachidonic acid (5 mg/mL) for 30 min at 37 °C. To investigate the effect of activated platelets on cellular uptake, we pre-incubated A549 cells with arachidonic acid-activated platelets for 30 min before incubation with HAS-MPs or anti-CD41-HSA-MPs for an additional 24 h (MPs/Platelets/A549 = 5000/250/1). The samples with or without Trypan blue quenching allow us to differentiate and quantify the fluorescent particles that have been completely engulfed versus those that are merely adhering to the cell membrane. Typan blue quenches the fluorescence of the HAS-MPs left on the surface of the cells. The flow cytometer quantified the cellular uptake percentage and MFI of A549 cells.

### 3.10. Validation of CD62P Ligand Expression Level in TCGA and CCLE Databases

CD62P binds to several common ligands, including CD44, SELPLG, and CD24. The Cancer Cell Line Encyclopedia (CCLE) project (accessed on 6 July 2022, https://www.broadinstitute.org/ccle) was used to validate the expression profiles of *CD44* and *SELPLG* in 1091 cell lines. The Cancer Genome Atlas (TCGA) database (accessed on 7 July 2022, https://portal.gdc.cancer.gov/) was used to validate the CD44 and SELPLG expression in 33 cancers. Different gene expression analysis was performed using the limma package in R studio software to determine whether CD44 or SELPLG expression varied between tumors and normal tissues.

### 3.11. Statistical Analysis

In addition to the statistical methods mentioned above, Student’s *t*-test was used to explore the association between the groups. Data were expressed as mean ± standard deviations (*SD*). * *p* < 0.05, ** *p* < 0.01, and *** *p* < 0.001 were thought to be statistically significant.

## 4. Conclusions

In this study, anti-CD41-HSA-MPs were designed to target CTCs by hitchhiking on platelets to track tumor cells. CD62P-HSA-MPs were used to adhere the tumor cells by taking advantage of the conjugation of CD62P. Both resultant modified HSA-MPs demonstrated the tumor cell-targeting ability in vitro. In summary, the platelet-based and platelet-mimicking human serum albumin submicron particles offered a promising approach for metastatic cancer therapy and tracking through accurate targeting for tumor cells.

## Figures and Tables

**Table 1:** Particle size and zeta potential.

Sample	Average Size (nm)	Polydispersity Index (PDI)	Zeta Potential (mV)	Conductivity (mS/cm)
anti-CD41-HSA-MPs	952 ± 41	0.24 ± 0.03	−25 ± 1	1.3 ± 0.1
CD62P-HSA-MPs	974 ± 15	0.26 ± 0.11	−24 ± 2	1.4 ± 0.1
HSA-MPs	847 ± 24	0.26 ± 0.11	−26 ± 1	1.3 ± 0.1

## Data Availability

The data that support the findings of this study are available from the corresponding author upon reasonable request. The datasets generated and analyzed during the current study are available in the TCGA database (https://portal.gdc.cancer.gov/) and the CCLE database (https://www.broadinstitute.org/ccle).
